# Molecular Testing in Ovarian Tumours: Challenges from the Pathologist’s Perspective

**DOI:** 10.3390/diagnostics13122072

**Published:** 2023-06-15

**Authors:** Kate Dinneen, Rupali Arora

**Affiliations:** Department of Cellular Pathology, University College London NHS Trust, 60 Whitfield Street, London W1T 4E, UK; kate.dinneen@nhs.net

**Keywords:** ovarian cancer, molecular diagnostics, histopathology

## Abstract

The use of molecular testing to direct diagnosis and treatment options in ovarian tumours has rapidly expanded in recent years, in particular with regard to the recommendation for routine homologous recombination deficiency (HRD) testing in all patients with high-grade ovarian epithelial tumours. The implications of this increased level of testing upon the pathologist is significant in terms of increased workload, the provision of adequate tumour samples for molecular testing, and the interpretation of complex molecular pathology reports. In order to optimise the quality of reports generated, it is important to establish clear pathways of communication on both a local and national level between clinicians, pathology lab staff, and medical scientists. On a national level, in the United Kingdom, Genomic Laboratory Hubs (GLHs) have been established to provide a uniform high-quality molecular diagnostics service to all patients with ovarian tumours within the National Health services in the country. On a local level, there are a number of small steps that can be taken to improve the quality of tissues available for testing and to streamline the processes involved in generating requests for molecular testing. This article discusses these factors from the perspective of the clinical histopathologist.

## 1. Introduction

According to the Global Cancer Statistics 2020, ovarian cancers are the eight most-common malignancy in women, accounting for 3.4% of all new cancer diagnoses in women worldwide [1]. Ovarian tumours comprise a diverse group of neoplasms, benign, malignant, and of intermediate malignant potential. In the most-recent fifth edition of the WHO classification of tumours, they are broadly subdivided according to their cell of origin into epithelial, mesenchymal, sex cord stromal, germ cell, and miscellaneous tumours. The epithelial tumours comprise the vast majority of malignant diagnoses, of which there are five main subgroups: serous, mucinous, endometrioid, clear cell, and Brenner. High-grade serous carcinoma (HGSC) remains the most-common histotype of all ovarian cancers [2].

The standard treatment for ovarian cancer is cytoreductive surgery followed by platinum-based chemotherapy; however, due in part to the high intratumoural heterogeneity of these neoplasms, most patients will eventually develop resistance to platinum-based therapies [3]. In light of the increasing knowledge of specific molecular alterations present in these tumours, it is expected that personalised targeted therapies will provide patients with better survival outcomes with reduced morbidity. At present, the number of potential molecular targets described in the literature far outstrips the availability of efficacious targeted therapies available in the clinical setting; however, with greater knowledge of targetable mutations and molecular pathways, it is highly likely that this ratio will begin to shift. Accordingly, it is imperative to establish clear guidelines and pathways for the implementation of molecular testing in the clinical setting. At present, in the United Kingdom, there is no established guideline for molecular testing in ovarian cancers. In the clinical setting, molecular testing is currently only performed as part of routine diagnostics in HGSC, endometrioid carcinoma, and clear cell carcinoma of the ovary (Table 1).

All patients with a new diagnosis of high-grade ovarian epithelial cancer (HGSC, grade-2–3 endometrioid carcinoma and clear cell carcinoma) are consented for homologous recombination deficiency (HRD) testing. Homologous recombination is a pathway involved in repairing double-strand DNA breaks, and therefore, its deficiency results in genomic instability, which is a target for PARP inhibitors [4]. HRD can be caused by germline or somatic mutations of genes involved in this pathway, of which BRCA genes are the most-commonly affected [5]. Approximately 50% of patients with HGSC have HRD, of which half are attributable to BRCA 1/2 mutations: 11–15% are germline mutations, and a further 5–7% are somatic [6,7]. The remaining cases comprise mutations in alternative genes within this pathway, calculated as a genomic instability score (GIS). The identification of HRD is of great significance for the patient as it not only informs prognosis and treatment options, but it can also prompt steps for the prevention of secondary malignancies, and in the case of germline mutations, it can lead to reflex testing of family members for inherited mutations. HRD testing is performed on tumour DNA and, therefore, cannot identify which BRCA mutations are germline. For this reason, HRD testing is performed in parallel with germline BRCA testing on blood samples. In the case of recurrent high-grade ovarian carcinoma, which has previously responded well to platinum chemotherapy, if the tumour shows HRD positivity, a PARP inhibitor can be used unless the patient has previously been treated with a PARP inhibitor.

Mismatch repair (MMR) deficiency testing is performed routinely in ovarian endometrioid and clear cell carcinomas, both of which are commonly associated with endometriosis [8,9]. The testing is most commonly performed using routine immunohistochemical (IHC) stains, and a deficiency can indicate a potential role for immune checkpoint inhibitor therapies, as well as prompt investigation for Lynch syndrome [9]. An alternative method of testing for MMR deficiency is through microsatellite instability (MSI) testing, which can be performed using polymerase chain reaction (PCR) or next-generation sequencing (NGS). This test assesses the lengths of a set of microsatellites in a tumour, which are short repetitive DNA sequences, which may expand or contract in length in the setting of microsatellite instability. Tumours are designated MSI high (MSI-H) if they show ≥2 unstable markers, MSI low (MSI-L) if they show 1 unstable marker, and microsatellite-stable (MSS) if there are 0 unstable markers. MMR testing by IHC and MSI molecular testing are considered to show a high concordance and, therefore, may be used interchangeably, depending on the preference of each individual laboratory [10]; however, MMR testing by IHC is a more inexpensive option and, therefore, often favoured. Similar to the molecular classification of endometrial endometrioid carcinomas, four molecular subgroups have been proposed for ovarian endometrioid carcinomas: MMR-deficient, POLE-mutated, TP53-mutated, and no specific molecular profile [11]. Whilst not currently employed in routine practice, consideration should be given to routine molecular testing of these tumours for the purposes of prognostication: MMR and POLE mutations are associated with favourable outcomes, whilst TP53-mutated tumours portend a worse prognosis.

Over 97% of HGSCs harbour a TP53 mutation, which is assessed at diagnosis using routine immunohistochemistry [12]. Mutation pattern P53 staining by IHC is seen in >95% of HGSCs; however, a small percentage of cases may be missed by this means of testing [13]. If a tumour shows morphology suggestive of HGSC, but wild-type P53 staining, or if it is necessary to use molecular testing to distinguish between HGSC and low-grade serous carcinoma (LGSC) in cases with overlapping morphologies, P53 testing can be performed using NGS to aid in this distinction. Similarly, although not required for diagnosis, TP53 mutations are commonly seen in both epithelial and sarcomatous components of carcinosarcomas, supporting a monoclonal epithelial origin [14].

Mucinous ovarian carcinomas are associated with the copy number loss of CDKN2A and KRAS mutations, both of which are believed to be early events in tumorigenesis as they have been identified in benign and borderline mucinous precursor neoplasms [15,16]. The identification of such mutations is not routinely required for diagnosis; however, it can be of great help in distinguishing these tumours from endometrioid ovarian adenocarcinoma with mucinous differentiation and from mucinous metastasis from distant sites, both of which are managed differently in the clinical setting.

Molecular testing is additionally performed in the clinical setting on an as-required basis in cases that pose diagnostic challenges, most notably in the sex cord stromal tumours. Almost all adult granulosa cell tumours harbour a recurrent somatic FOXL2 point mutation [17], whilst Sertoli cell tumours and Sertoli–Leydig cell tumours are associated with somatic DICER1 mutations [18,19,20]. The rare sex cord tumour with annular tubules (SCTATs) is most commonly associated with Peutz–Jegher syndrome (PJS), although a subset of these tumours is non-syndromic and is associated with a worse prognosis [21]. For this reason, in cases without a pre-existing diagnosis of PJS, the demonstration of an STK11 germline mutation is of benefit in terms of both prognostication and ongoing broader clinical care [22]. Small cell carcinoma of the ovary, hypercalcaemic type, is an aggressive tumour, which typically poses diagnostic challenges; reaching this diagnosis can be aided by the demonstration of a somatic or germline SMARCA4 mutation [23]. Similarly, in cases where the distinction between a benign leiomyoma and its malignant counterpart leiomyosarcoma is difficult, the demonstration of an MED12 mutation can be of great assistance in confirming benignity [24].

A further subset of ovarian tumours has molecular alterations described in the literature; however, these are not required for diagnosis and have not as of yet been proven to be efficacious in the clinical setting in terms of directing targeted therapies. For example, low-grade serous carcinomas with KRAS or BRAF mutations are associated with a favourable prognosis; however, these are not routinely tested [25]. Similarly, mutations in CDKN2A and KRAS are commonly found in mucinous ovarian carcinomas as an early event in tumourigenesis, but are not required for diagnosis [26]. Furthermore, a subset of germ cell tumours can acquire KRAS, KIT, and BRCA mutations and have been associated with alterations in chromosome 12 [26,27,28,29].

It is, therefore, evident that the landscape for molecular testing in ovarian tumours is likely to change dramatically in the coming years as targeted therapies become available for as-of-yet unactioned tumour mutations. In an attempt to better organise molecular testing within the United Kingdom and to ensure equitable access to all patients, genomic testing is now delivered via seven Genomic Laboratory Hubs (GLHs), which are distributed throughout the country, with each hub responsible for coordinating the molecular diagnostic services for its own region. Requests for molecular testing are submitted via online forms to increase efficiency; however, not all tests are currently performed within the NHS. At present, HRD testing is outsourced to Myriad Laboratory in the United States. This has an impact on turnaround times (TATs), as diagnostic material must be sent abroad for testing. The intention is to ultimately consolidate all molecular testing services to within the NHS, which will have a significant impact on reducing TATs and, therefore, enhance patient outcomes through the rapid generation of molecular reports.

In order to deliver a timely, streamlined, cost-effective, and accurate molecular diagnostic service, the pathologist plays a key role as an intermediary between the clinicians and those involved in molecular testing. The challenges posed by this process are plentiful and shall be discussed in detail below.

## 2. Considerations for Tissue Sampling and Interpretation

There are multiple steps involved in the generation of a timely and accurate molecular pathology report; therefore, it is imperative that clear pathways for the communication of requests for tumour testing are jointly established between clinical teams and pathology services at both local and national levels. The processes involved in molecular testing of any tumour type can be broken down into pre-analytical, analytical, and post-analytical phases. The pre-analytical phase starts with tissue sampling and extends up to the point of molecular testing, which is the analytical phase. The post-analytical phase refers to the interpretation and communication of test results. The pathologist is involved in both the pre- and post-analytical phases, whilst the molecular testing is performed by biomedical scientists and biostatisticians. There are a number of variables present within the pre- and post-analytical phases that can be modified and optimised in order to ensure the greatest chance of generating accurate test results, as discussed below.

## 3. Pre-Analytical Considerations for the Pathologist

**Figure 1:**
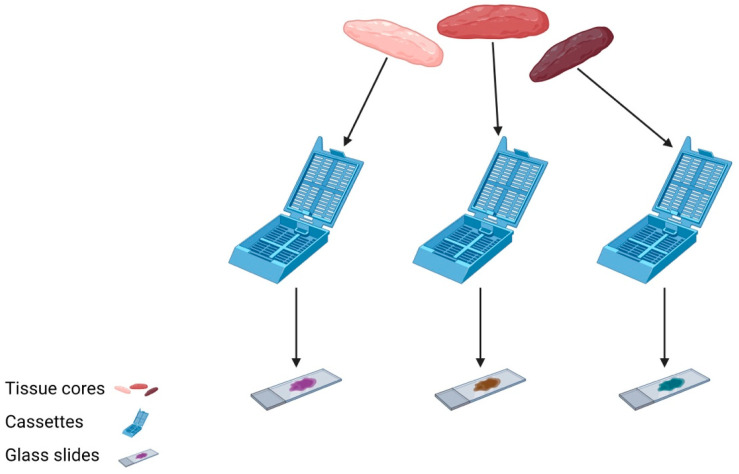
Three tissue cores are individually submitted in separate cassettes and processed into H&E sections for assessment prior to selecting material most-suitable for molecular analysis.

In order to generate a robust and accurate molecular pathology report, a sufficient volume of viable tumour cells is required for testing. When a tissue sample is processed in the lab, sections are first cut to assess tumour morphology on a routine H&E slide. In many instances, further sections are required for ancillary testing with immunohistochemistry (IHC) and/or special stains, to confirm or refine the diagnosis. It is often not until this point that the remaining tissue can be sent for molecular testing, if required. It is, therefore, apparent that scanty biopsy samples are not likely to contain sufficient tumour cells to both accurately identify the tumour histotype and perform appropriate molecular testing. For this reason, it is strongly recommended that more than one tissue core is taken when performing diagnostic biopsy sampling. In order to preserve tissue for molecular testing, individual cores can be embedded into separate blocks, and a single H&E will be processed for each in order to assess morphology in the first instance (Figure 1). Based on microscopic appearances, one block will be chosen for molecular testing and a separate block will be used for further ancillary testing, thus optimising the available material.

Intratumoural heterogeneity plays an important role in influencing the results of molecular tests. Ovarian cancers are known to have high rates of heterogeneity, which refers to the fact that there is a phenotypically diverse population of cancer cells present both within a single tumour and across multiple different tumour subtypes [30]. This diversity is thought to play an important role in the development of treatment resistance [31] and may lead to the failure of personalised medical therapies if only a single subpopulation of tumour cells is sampled and tested, thus underestimating the molecular landscape of the tumour [32]. The ability of the pathologist to avoid these sampling biases is based on the assessment of tumour morphology on H&E. Within a single tumour, certain areas may show different morphologies, each of which may potentially harbour a different molecularly defined subpopulation of tumour cells. By taking more than one biopsy core at the time of tissue sampling, the pathologist can analyse the morphological appearances of each area of tumour sampled and select the most-appropriate core in order to provide the most-representative material for molecular analysis.

Each molecular test has a pre-defined limit of detection, which refers to the lowest concentration of analyte that can be detected and consistently generate an accurate test result. The analyte in question in the setting of molecular testing in ovarian cancer is the tumour cell, and the concentration is the number of tumour cell nuclei divided by the total number of nuclei present in a tissue sample. A high-quality sample should contain a percentage of tumour cell nuclei that measures at least double the stated limit of detection for the required test. In general, this corresponds to ≥30% tumour cell nuclei; however, >20% will often suffice. It is clear that tissue samples with large numbers of background benign cells, including inflammatory cells and stromal cells, will result in a reduction in the percentage of tumour cell nuclei present for assessment; however, steps can be taken by the pathologist to avoid this becoming an issue. By examining the morphology of each tumour sample on H&E prior to requesting molecular tests, it is possible to choose the sample with the greatest volume of tumour cell nuclei upfront to avoid inconclusive or failed test results. Similarly, one can macro-dissect an area of tissue with high tumour cell content away from the surrounding tissue and send this alone for molecular testing in order to reduce the impact of non-tumoural tissue on the molecular report generated.

**Figure 2:**
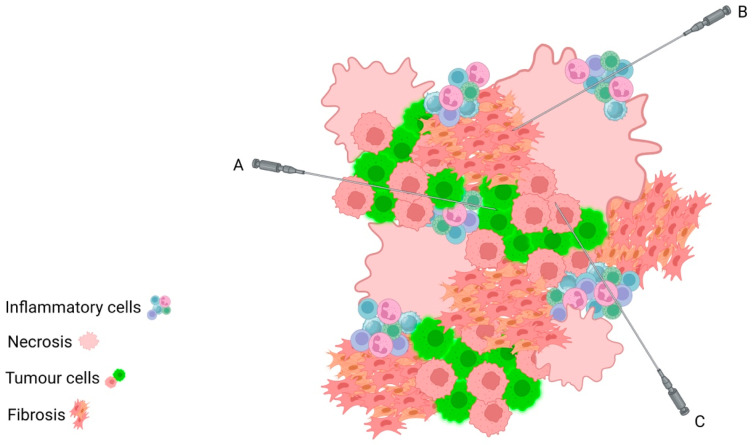
Schematic depicting intratumoural heterogeneity, with areas of necrosis, fibrosis, and inflammation admixed amongst tumour cells. Despite sampling from the same tumour mass, Core Biopsy A will sample an area of almost pure tumour cells; Core Biopsy B will sample background stromal and necro inflammatory cells only; Core Biopsy C will sample a mix of inflammatory cells and tumour cells. This highlights the importance of taking multiple core biopsies in order to ensure that the tumour cells are adequately sampled for assessment.

It is very difficult to macroscopically predict the tumour cell content of any tissue at the time of sampling; however, there are a number of factors to consider that may indicate a lower quality sample site. Many tumours will contain areas of necrosis, which can often be identified macroscopically. These areas are to be avoided when taking biopsy samples as they do not contain viable tumour cells. In the setting of percutaneous biopsy sampling, it may be impossible to avoid these areas, thus multiple biopsies should be taken to avoid the need for a repeat procedure (Figure 2). Similarly, it is important to be mindful that, in the post-neoadjuvant chemotherapy (NACT) setting, patients who have had a good response to treatment will have replaced tumour cells with areas of fibrosis, inflammation, benign reactive stromal cells, and necrosis, thus reducing the number of viable tumour cells. The use of electrocoagulation when sampling tissues may also lead to tissue damage, may complicate the interpretation of the tumour morphology, and may reduce the number of tumour cells suitable for analysis. The practice of taking multiple tissue cores helps to avoid these diagnostic issues as the likelihood of obtaining a core that contains sufficient tumour cells is dramatically increased.

There is a clear hierarchy in terms of the best type of material for molecular testing. Resection specimens that have not received NACT are theoretically ideal as they classically provide large volumes of viable tumour for testing; however, this is not often the tissue type chosen for analysis in light of both clinical need for a pre-operative tissue diagnosis and the frequency of standard neoadjuvant treatment regimens currently administered. A good-quality biopsy sample is often the preferred choice, as it may be used to direct delivery of neoadjuvant therapies. As previously discussed, it is imperative to take more than one biopsy at the time of sampling. The use of post-NACT tissue for analysis is often suboptimal, depending on the degree of response to treatment seen, as defined by the chemotherapy response score (CRS). A CRS score of 1 refers to no or minimal response to therapy; CRS 2 describes an appreciable response to therapy with readily identifiable residual tumour; CRS 3 is a complete or near complete response to therapy, with scattered residual tumour cells remaining at most [33]. The greater the volume of residual tumour cells, the greater the likelihood of reaching the limit of detection and, thus, providing sufficient material to generate a reliable molecular pathology report. Finally, cytology samples can be used for molecular testing if they are first converted into a cell block; however, it is not currently possible to provide robust molecular testing on liquid-based samples. Other samples that are not suitable for molecular testing include decalcified samples such as bone metastases.

Once a tissue sample has been taken from a patient, it is routine practice for it to be placed immediately into 10% neutral buffered formalin, following which it is processed and embedded in paraffin wax to create a formalin-fixed paraffin-embedded (FFPE) block [34]. The optimal fixation time prior to processing will be influenced by different factors including tissue type and specimen size; however, the current recommendations are that a fixation time of 6–24 h is optimal. Whilst a tissue fixation time of over 24 h may either enhance or have no impact on tissue morphology, excessive formalin fixation can impair DNA quality for molecular testing due to DNA damage. This damage can be induced by a number of different mechanisms, including DNA fragmentation and the generation of formaldehyde-induced crosslinks between proteins and proteins, DNA and DNA, or DNA and proteins within the tissue [35,36]. By contrast, tissue under fixation may impair both morphological appearances and also negatively impact DNA preservation for molecular analysis.

The benefit of adequate tissue fixation prior to processing is that the tissues are preserved in as life-like a state as possible through inhibition of autolysis. This is of particular importance in the setting of surgeries or biopsy clinics held on a Friday afternoon: because most pathology laboratories do not operate a full service over the weekend, if a sample is not placed in formalin at the time of removal by the clinical team, it is possible that this will not be recognised over the weekend. In this case, the degree of natural autolysis that the tissue will undergo may lead to inadequate viable tumour cells available for both histological and molecular diagnostics. Similarly, as is often seen in institutions with pathology departments located remote from surgical theatres, delays in the transportation of specimens to the lab following retrieval may result in tissue over-fixation, with inherent potential for an impact on DNA quality.

Not all material that has been formalin-fixed will reliably provide high-quality DNA for molecular analysis. In many instances, as briefly mentioned above, the DNA extracted from FFPE tissue is often fragmented and of poor quality as a result of the process of fixation and on a molecular level may contain artefactual sequence alterations resulting from crosslinking of protein molecules [37]. Nevertheless, formalin remains to date the most-appropriate fixative solution available. Currently, fresh tissue is not used for molecular analysis in ovarian tumours, and material used for frozen section may be used after it has been thawed and processed into an FFPE block. The age of the sample is also of importance, as older archival samples are less likely to yield high-quality DNA samples, as compared to DNA extracted from more recently prepared FFPE tissues.

When a tissue diagnosis that prompts further molecular testing has been made by a pathologist based on morphological appearances, the process of requesting molecular testing depends on the test type and indication. In the setting of HRD testing for HGSC, patients must first be consented by the clinical team, and this consent (or lack thereof) should clearly be stated on the histology request form or in another predetermined location agreed by those involved in this care pathway. Tests that are performed as part of routine diagnostics do not require such explicit consent. Following the identification of the most-appropriate tumour sample, tissue curls of a 6 μm thickness should be cut by the medical scientists in the pathology laboratory and sent along with the completed molecular request form to the appropriate GLH to which the referring hospital is connected. Providing tissue curls, as opposed to sections mounted onto glass slides, is preferrable as they result in less damage to tissues during DNA extraction because the tissues do not need to be scraped off of slides. It is worth noting that the preferences of different molecular laboratories may vary in terms of how they wish to receive and process the material; thus, conferring with the GLH prior to sending specimens is advised. The pathologist can help to expedite molecular testing, for example in the case of HRD, by requesting tissue sections or curls reflexively upon making a diagnosis of HGSC of stage 3 and above. Once patient consent has been confirmed, the lab can release this material to the GLH for testing. This process can be made yet more efficient by communicating patient consent via electronic patient records, rather than through emails between clinicians and pathologists.

From this point on, the process of molecular analysis enters into the analytical phase, which is regularly performed by biomedical scientists and biostatisticians rather than histopathologists.

## 4. Post-Analytical Considerations for the Pathologist

Following molecular testing, the pathologist is furnished with a molecular report from the GLH, which needs to be interpreted in the clinical context of the individual patient and communicated to the clinical team in an efficient, clear, and concise manner. This is often a non-onerous task in the case of straightforward reports; however, it becomes more difficult when the results generated are more complex and require a greater level of understanding of molecular biology to interpret. It is important remember that most histopathologists working in the clinical setting do not have specific training in high-level molecular pathology. There is now a clear career path for those who wish to pursue this route, with FRCPath fellow status by examination; however, this route is normally pursued by medical scientists and biostatisticians, rather than clinical histopathologists. It is, therefore, imperative that a clear report is provided to the pathologist in the first instance so that there are no interpretive errors or delays in generating a report to send to the clinical teams.

The time commitments involved in issuing molecular reports has also increased significantly in recent years, as both the availability of and indications for molecular testing have expanded rapidly. Previously, molecular testing in the case of HGSC was limited primarily to patients with a known significant family history, whilst at present, HRD testing is performed in every patient who provides consent as a positive result can guide the delivery of targeted therapies. This results in a far greater number of tests that must be requested, interpreted, and reported by the pathologist, all with their own inherent time commitments. Similarly, this increased testing imparts an economic impact on the pathology services. On a local level, many of these costs have been addressed by the establishment of national GLHs, which perform the tests; however, increased expenses relating to tissue processing, transport, and storage are generally absorbed by the local department.

## 5. Conclusions

The use of molecular pathology in the clinical setting has changed dramatically in recent years and is set to transform yet again in the near future as our understanding of the molecular biology of ovarian tumours increases and as new targeted medical therapies are developed. Due to the significant potential impact on the treatment and broader management of individual patients and their families posed by the results of these tests, it is imperative that all steps are taken in order to ensure that the testing has the greatest chance of success. The pathologist is heavily involved in the process of molecular testing; however, as discussed above, their ability to provide high-quality tissue samples for testing is heavily influenced by various preanalytical factors. It is advised that clear pathways for communication of molecular test requests be established on a local and national level, with regular feedback between the teams regarding current issues with the process. The establishment of small changes to practice, as required, can have an enormous impact on the quality of test results provided and, therefore, greatly optimise the clinical outcomes for patients with ovarian tumours under our care.

**Table 1:** Ovarian tumours with associated molecular abberrations.

Tumour Type	Associated Molecular Aberrations
**Epithelial Tumours**	
High-grade serous carcinoma [7,12,38]	TP53 * HRD * Germline BRCA 1/2 *
Low-grade serous carcinoma [39]	KRAS BRAF NRAS
Mucinous carcinoma [26]	CDKN2A KRAS TP53 HER2 amplification
Endometrioid carcinoma [40,41,42]	MMR * CTNNB1 PIK3CA PTEN KRAS ARID1A POLE TP53
Clear cell carcinoma [8,42,43,44,45]	MMR * ARID1A PIK3CA TERT KRAS TP53
Brenner tumour [46]	Key molecular alterations not identified
Mesonephric-like adenocarcinoma [47,48]	PIK3CA KRAS NRAS
Carcinosarcoma [14]	TP53 *
**Sex Cord Stromal Tumours**	
Adult granulosa cell tumour [17]	FOXL2 (somatic) **
Juvenile granulosa cell tumour [49,50,51]	AKT1 GNAS IDH 1/2
Sertoli cell tumour [18]	DICER1 (somatic) **
Sertoli–Leydig cell tumour [19,20,52]	DICER1 (somatic) ** FOXL2
Sex cord tumour with annular tubules (SCTATs) [22]	STK11 **
**Germ Cell Tumours**	
Yolk sac tumour [27]	Chromosome 12 abnormalities
Dysgerminoma [26]	Chromosome 12 abnormalities KIT
Embryonal carcinoma [28]	Chromosome 12 abnormalities
**Mesenchymal Tumours**	
Leiomyoma [24]	MED12 **
**Miscellaneous Tumours**	
Small cell carcinoma of ovary, hypercalcaemic type [23]	SMARCA4 (somatic or germline) **

* Used in routine clinical practice. ** Used in clinical practice to aid diagnosis in difficult cases.

## Data Availability

No new data was created.

## References

[1] Sung H., Ferlay J., Siegel R.L., Laversanne M., Soerjomataram I., Jemal A., Bray F. (2021). Global Cancer Statistics 2020: GLOBOCAN Estimates of Incidence and Mortality Worldwide for 36 Cancers in 185 Countries. CA Cancer J. Clin..

[2] Kim J., Park E.Y., Kim O., Schilder J.M., Coffey D.M., Cho C.-H., Bast R.C. (2018). Cell Origins of High-Grade Serous Ovarian Cancer. Cancers.

[3] Guan L., Lu Y. (2018). New developments in molecular targeted therapy of ovarian cancer. Discov. Med..

[4] Ledermann J.A., Drew Y., Kristeleit R.S. (2016). Homologous recombination deficiency and ovarian cancer. Eur. J. Cancer.

[5] Fuh K., Mullen M., Blachut B., Stover E., Konstantinopoulos P., Liu J., Matulonis U., Khabele D., Mosammaparast N., Vindigni A. (2020). Homologous recombination deficiency real-time clinical assays, ready or not?. Gynecol. Oncol..

[6] Frey M.K., Pothuri B. (2017). Homologous recombination deficiency (HRD) testing in ovarian cancer clinical practice: A review of the literature. Gynecol. Oncol. Res. Pract..

[7] Cancer Genome Atlas Research Network (2011). Integrated genomic analyses of ovarian carcinoma. Nature.

[8] Wiegand K.C., Shah S.P., Al-Agha O.M., Zhao Y., Tse K., Zeng T., Senz J., McConechy M.K., Anglesio M.S., Kalloger S.E. (2010). ARID1A mutations in endometriosis-associated ovarian carcinomas. N. Engl. J. Med..

[9] Fraune C., Rosebrock J., Simon R., Hube-Magg C., Makrypidi-Fraune G., Kluth M., Büscheck F., Höflmayer D., Schmalfeldt B., Müller V. (2020). High homogeneity of MMR deficiency in ovarian cancer. Gynecol. Oncol..

[10] Dedeurwaerdere F., Claes K.B., Van Dorpe J., Rottiers I., Van der Meulen J., Breyne J., Swaerts K., Martens G. (2021). Comparison of microsatellite instability detection by immunohistochemistry and molecular techniques in colorectal and endometrial cancer. Sci. Rep..

[11] Parra-Herran C., Lerner-Ellis J., Xu B., Khalouei S., Bassiouny D., Cesari M., Ismiil N., Nofech-Mozes S. (2017). Molecular-based classification algorithm for endometrial carcinoma categorizes ovarian endometrioid carcinoma into prognostically significant groups. Mod. Pathol..

[12] Ahmed A.A., Etemadmoghadam D., Temple J., Lynch A.G., Riad M., Sharma R., Stewart C., Fereday S., Caldas C., Defazio A. (2010). Driver mutations in TP53 are ubiquitous in high grade serous carcinoma of the ovary. J. Pathol..

[13] Kim H.N., Woo H.Y., Do S.I., Kim H.S. (2019). Targeted sequencing of tubo-ovarian and peritoneal high-grade serous carcinoma with wild-type p53 immunostaining pattern. In Vivo.

[14] Jin Z., Ogata S., Tamura G., Katayama Y., Fukase M., Yajima M., Motoyama T. (2003). Carcinosarcomas (Malignant Mullerian Mixed Tumors) of the Uterus and Ovary: A Genetic Study With Special Reference to Histogenesis. Int. J. Gynecol. Pathol..

[15] De Leo A., Santini D., Ceccarelli C., Santandrea G., Palicelli A., Acquaviva G., Chiarucci F., Rosini F., Ravegnini G., Pession A. (2021). What Is New on Ovarian Carcinoma: Integrated Morphologic and Molecular Analysis Following the New 2020 World Health Organization Classification of Female Genital Tumors. Diagnostics.

[16] Cheasley D., Wakefield M.J., Ryland G.L., Allan P.E., Alsop K., Amarasinghe K.C., Ananda S., Anglesio M.S., Au-Yeung G., Böhm M. (2019). The molecular origin and taxonomy of mucinous ovarian carcinoma. Nat. Commun..

[17] Shah S.P., Köbel M., Senz J., Morin R.D., Clarke B.A., Wiegand K.C., Leung G., Zayed A., Mehl E., Kalloger S.E. (2009). Mutation of FOXL2 in granulosa-cell tumors of the ovary. N. Engl. J. Med..

[18] Conlon N., Schultheis A.M., Piscuoglio S., Silva A., Guerra E., Tornos C., E Reuter V., A Soslow R., Young R.H., Oliva E. (2015). A survey of DICER1 hotspot mutations in ovarian and testicular sex cord-stromal tumors. Mod. Pathol..

[19] de Kock L., Terzic T., McCluggage W.G., Stewart C.J., Shaw P., Foulkes W.D., Clarke B.A. (2017). DICER1 Mutations Are Consistently Present in Moderately and Poorly Differentiated Sertoli-Leydig Cell Tumors. Am. J. Surg. Pathol..

[20] Wang Y., Chen J., Yang W., Mo F., Senz J., Yap D., Anglesio M.S., Gilks B., Morin G.B., Huntsman D.G. (2015). The Oncogenic Roles of DICER1 RNase IIIb Domain Mutations in Ovarian Sertoli-Leydig Cell Tumors. Neoplasia.

[21] Young R.H., Welch W.R., Dickersin G.R., Scully R.E. (1982). Ovarian sex cord tumor with annular tubules: Review of 74 cases including 27 with Peutz-Jeghers syndrome and four with adenoma malignum of the cervix. Cancer.

[22] Tan H., Mei L., Huang Y., Yang P., Li H., Peng Y., Chen C., Wei X., Pan Q., Liang D. (2016). Three novel mutations of STK11 gene in Chinese patients with Peutz–Jeghers syndrome. BMC Med. Genet..

[23] Jelinic P., Mueller J.J., Olvera N., Dao F., Scott S.N., Shah R., Gao J., Schultz N., Gonen M., A Soslow R. (2014). Recurrent SMARCA4 mutations in small cell carcinoma of the ovary. Nat. Genet..

[24] Li Z., Maeda D., Kudo-Asabe Y., Tamura D., Nanjo H., Hayashi A., Ikemura M., Fukayama M., Goto A. (2018). MED12 is frequently mutated in ovarian and other adnexal leiomyomas. Hum. Pathol..

[25] Kaldawy A., Segev Y., Lavie O., Auslender R., Sopik V., Narod S.A. (2016). Low-grade serous ovarian cancer: A review. Gynecol. Oncol..

[26] Cheng L., Roth L.M., Zhang S., Wang M., Morton M.J., Zheng W., Karim F.W.A., Montironi R., Lopez-Beltran A. (2010). KIT gene mutation and amplification in dysgerminoma of the ovary. Cancer.

[27] Riopel M.A., Spellerberg A., Griffin C.A., Perlman E.J. (1998). Genetic analysis of ovarian germ cell tumors by comparative genomic hybridization. Cancer Res..

[28] Cheng L., Zhang S., Talerman A., Roth L.M. (2010). Morphologic, immunohistochemical, and fluorescence in situ hybridization study of ovarian embryonal carcinoma with comparison to solid variant of yolk sac tumor and immature teratoma. Hum. Pathol..

[29] Cheung A., Shah S., Parker J., Soor P., Limbu A., Sheriff M., Boussios S. (2022). Non-Epithelial Ovarian Cancers: How Much Do We Really Know?. Int. J. Environ. Res. Public Health.

[30] Kossaï M., Leary A., Scoazec J.Y., Genestie C. (2018). Ovarian cancer: A heterogeneous disease. Pathobiology.

[31] Prasetyanti P.R., Medema J.P. (2017). Intra-tumor heterogeneity from a cancer stem cell perspective. Mol. Cancer.

[32] Gerlinger M., Rowan A.J., Horswell S., Larkin J., Endesfelder D., Gronroos E., Martinez P., Matthews N., Stewart A., Tarpey P. (2012). Intratumor heterogeneity and branched evolution revealed by multiregion sequencing. N. Engl. J. Med..

[33] Lawson B.C., Euscher E.D., Bassett R.L., Liu J., Ramalingam P., Zhong Y., Fleming N.D., Malpica A. (2020). A Three-Tier Chemotherapy Response Score For Ovarian/Fallopian Tube/Peritoneal High Grade Serous Carcinoma, Is It Clinically Relevant?. Am. J. Surg. Pathol..

[34] Sadeghipour A., Babaheidarian P. (2018). Making formalin-fixed, paraffin embedded blocks. Biobanking.

[35] McDonough S.J., Bhagwate A., Sun Z., Wang C., Zschunke M., Gorman J.A., Kopp K.J., Cunningham J.M. (2019). Use of FFPE-derived DNA in next generation sequencing: DNA extraction methods. PLoS ONE.

[36] Do H., Dobrovic A. (2015). Sequence artifacts in DNA from formalin-fixed tissues: Causes and strategies for minimization. Clin. Chem..

[37] Wallace A.J. (2016). New challenges for BRCA testing: A view from the diagnostic laboratory. Eur. J. Hum. Genet..

[38] Verhaak R.G., Tamayo P., Yang J.-Y., Hubbard D., Zhang H., Creighton C.J., Fereday S., Lawrence M., Carter S.L., Mermel C.H. (2012). Prognostically relevant gene signatures of high-grade serous ovarian carcinoma. J. Clin. Investig..

[39] Hunter S.M., Anglesio M.S., Ryland G.L., Sharma R., Chiew Y.-E., Rowley S.M., Doyle M.A., Li J., Gilks C.B., Moss P. (2015). Molecular profiling of low grade serous ovarian tumours identifies novel candidate driver genes. Oncotarget.

[40] Wu R., Hendrix-Lucas N., Kuick R., Zhai Y., Schwartz D.R., Akyol A., Hanash S., Misek D.E., Katabuchi H., Williams B. (2007). Mouse Model of Human Ovarian Endometrioid Adenocarcinoma Based on Somatic Defects in the Wnt/β-Catenin and PI3K/Pten Signaling Pathways. Cancer Cell.

[41] McConechy M.K., Ding J., Senz J., Yang W., Melnyk N., A Tone A., Prentice L.M., Wiegand K.C., McAlpine J.N., Shah S.P. (2013). Ovarian and endometrial endometrioid carcinomas have distinct CTNNB1 and PTEN mutation profiles. Mod. Pathol..

[42] Campbell I.G., Russell S.E., Choong D.Y.H., Montgomery K.G., Ciavarella M.L., Hooi C.S.F., Cristiano B.E., Pearson R.B., Phillips W.A. (2004). Mutation of the PIK3CA Gene in Ovarian and Breast Cancer. Cancer Res..

[43] Wu R.-C., Ayhan A., Maeda D., Kim K.-R., A Clarke B., Shaw P., Chui M.H., Rosen B., Shih I.-M., Wang T.-L. (2014). Frequent somatic mutations of the telomerase reverse transcriptase promoter in ovarian clear cell carcinoma but not in other major types of gynaecological malignancy. J. Pathol..

[44] Wang Y.K., Bashashati A., Anglesio M.S., Cochrane D.R., Grewal D.S., Ha G., McPherson A., Horlings H.M., Senz J., Prentice L.M. (2017). Genomic consequences of aberrant DNA repair mechanisms stratify ovarian cancer histotypes. Nat. Genet..

[45] Parra-Herran C., Bassiouny D., Lerner-Ellis J., Olkhov-Mitsel E., Ismiil N., Hogen L., Vicus D., Nofech-Mozes S. (2019). p53, mismatch repair protein, and POLE abnormalities in ovarian clear cell carcinoma: An outcome-based clinicopathologic analysis. Am. J. Surg. Pathol..

[46] Folkins A., Palacios J., Cheng X.W. (2020). WHO Classification of Tumours: Female Genital Tumours.

[47] Mirkovic J., McFarland M., Garcia E., Sholl L.M., Lindeman N., MacConaill L., Dong F., Hirsch M., Nucci M.R., Quick C.M. (2018). Targeted Genomic Profiling Reveals Recurrent KRAS Mutations in Mesonephric-like Adenocarcinomas of the Female Genital Tract. Am. J. Surg. Pathol..

[48] McCluggage W.G., Vosmikova H., Laco J. (2020). Ovarian combined low-grade serous and mesonephric-like adenocarcinoma: Further evidence for a Mullerian origin of mesonephric-like adenocarcinoma. Int. J. Gynecol. Pathol..

[49] Plevová P., Geržová H. (2019). Genetic Causes of Rare Pediatric Ovarian Tumors. Klin. Onkol..

[50] Kalfa N., Ecochard A., Patte C., Duvillard P., Audran F., Pienkowski C., Thibaud E., Brauner R., Lecointre C., Plantaz D. (2006). Activating mutations of the stimulatory g protein in juvenile ovarian granulosa cell tumors: A new prognostic factor?. J. Clin. Endocrinol. Metab..

[51] Bessière L., Todeschini A.-L., Auguste A., Sarnacki S., Flatters D., Legois B., Sultan C., Kalfa N., Galmiche L., Veitia R.A. (2015). A Hot-spot of In-frame Duplications Activates the Oncoprotein AKT1 in Juvenile Granulosa Cell Tumors. Ebiomedicine.

[52] Karnezis A.N., Wang Y., Keul J., Tessier-Cloutier B., Magrill J., Kommoss S., Senz J., Yang W., Proctor L., Schmidt D. (2019). DICER1 and FOXL2 Mutation Status Correlates With Clinicopathologic Features in Ovarian Sertoli-Leydig Cell Tumors. Am. J. Surg. Pathol..

